# Cardiovascular Risks of Hypertension: Lessons from Children with Chronic Kidney Disease

**DOI:** 10.3390/children9111650

**Published:** 2022-10-28

**Authors:** You-Lin Tain, Chien-Ning Hsu

**Affiliations:** 1 Division of Pediatric Nephrology, Kaohsiung Chang Gung Memorial Hospital, Kaohsiung 833, Taiwan; 2 College of Medicine, Chang Gung University, Taoyuan 333, Taiwan; 3 Department of Pharmacy, Kaohsiung Chang Gung Memorial Hospital, Kaohsiung 833, Taiwan; 4 School of Pharmacy, Kaohsiung Medical University, Kaohsiung 807, Taiwan

**Keywords:** cardiovascular disease, biomarkers, hypertension, chronic kidney disease, children, congenital anomalies of the kidney and urinary tract, ambulatory blood pressure monitoring, endothelial dysfunction

## Abstract

Hypertension is the most common complication of chronic kidney disease (CKD) in children, having a strong association with subsequential cardiovascular disease (CVD). In pediatric CKD, a considerable percentage of children with hypertension are undiagnosed or undertreated. Prior research has evaluated structural and functional markers of subclinical CVD and biomarkers in adults with CKD, while ideal biomarkers in pediatrics are still insufficiently studied. The ultimate goal of this review is to summarize what is currently known about state of hypertension, cardiovascular risk factors, and potential CVD markers/biomarkers in children with pre-dialysis CKD. We discuss omics-related biomarkers and the pathophysiologic processes of endothelial dysfunction, kidney injury, oxidative stress and inflammation that are classified by specific biomarkers. Moreover, we illustrate the existing challenges and highlight the paucity of pediatric CKD research to evaluate these CVD biomarkers for future clinical pediatric practice. Thus, achieving clinical utility of CVD biomarkers for use in pediatric CKD remains a significant challenge requiring additional efforts.

## 1. Introduction

Hypertension ranks as a leading modifiable risk factor for cardiovascular disease (CVD), not only in adults, but also in the youth [1,2]. A global meta-analysis to assess pediatric hypertension revealed the pooled prevalence of hypertension was 4.0% and its prevalence increased from 1.3% (1990–1999) to 6.0% (2010–2014) [3].

The most common type of hypertension in children is secondary hypertension, with chronic kidney disease (CKD) being the leading cause [4]. However, nowadays, primary hypertension is on the rise also in pediatric population [3,4]. In a large single-center study including 1025 hypertensive children, kidney diseases present two-thirds of total cases [5]. In children with CKD, hypertension is common with a range from 48% to 70% [6]. Of note is that more than 90% of hypertensive children are not receiving treatment [7]. Importantly, even if hypertensive children with CKD receive anti-hypertensive medication, up to 74% of them are still uncontrolled [6], not to mention that uncontrolled hypertension is a major risk factor for progression to end stage kidney disease (ESKD). Although increased CV events and accelerated atherosclerosis have been extensively documented in patients with ESKD, increasing evidence highlights the increased risk for CV events that prevails in early stages of CKD. Therefore, early detection and adequate management of hypertension in CKD youth should be prioritized, to advance their kidney and CV outcomes.

CV complications in CKD can be assigned to two different but associated mechanisms by so-called atherosclerosis and arteriosclerosis [8]. The former mainly affects conduit function, whereas the latter is characterized by arterial stiffness and hypertrophy of large conduit arteries [9]. Endothelial dysfunction is the clinical entity of atherosclerosis and is the initial event behind pathogenesis of CVD [10]. Several traditional (e.g., hypertension, diabetes, hyperlipidemia, etc.) and CKD-related risk factors (e.g., inflammation, mineral bone disorder, uremic toxin, etc.) for CVD are more prevalent in children with CKD [11]. Importantly, many of these factors are associated with endothelial dysfunction.

CVD biomarkers offer the potential for non-invasive vascular function tests and have been identified as surrogates for CV outcome [12,13]. The identification of CVD biomarkers allows for the classification of CKD children to appropriate CV risk groups. Newly discovered CVD biomarkers and knowledge about the CVD risk groups could aid in taking rapid therapeutic intervention aimed at limiting these CV risks.

So far, there is scant hypertension research to help address the existing knowledge-practice gaps in children with CKD. Less attention has been drawn on the BP measurement and identification of CVD biomarkers in pediatric CKD compared to adults. This review aims to summarize what is currently known about CV risks of hypertension in children with CKD, the existing challenges, and potential CVD biomarkers aimed at improving future cardiovascular and kidney health in pediatric CKD, with a focus on pre-dialysis CKD stages.

We retrieved the literature from all of the articles published in English between January 1980 and August 2022 that were indexed in the MEDLINE/PubMed or EMBASE databases. We used following list of keywords: “hypertension”, “blood pressure”, “chronic kidney disease”, “congenital anomalies of the kidney and urinary tract”, “cardiovascular disease”, “arterial stiffness”, “atherosclerosis”, “endothelial dysfunction”, “biomarker”, “omics”, “children”, “pediatric”, “childhood”, “inflammation”, “nitric oxide”, “oxidative stress”, “risk factor”, “ambulatory blood pressure monitoring”, “left ventricular mass index”, “pulse wave velocity”, “carotid intima-media thickness”, and “uremic toxin”. Additional studies were then selected and evaluated according to references from eligible articles. We found that there are more than 5000 publications related to CVD risk factors/markers and CKD. However, less than 5% belong to pediatric research. We included both positive and negative studies. Moreover, both original research and review articles were included. Among them, original articles accounts for nearly 80% of searchable publications. Letters to the Editor, study protocols and conference proceedings were excluded. In total, we screened 203 full-text reports for eligibility. Our search showed that there is a relative dearth of information on CV risk factors and biomarkers in pediatric CKD.

## 2. State of Hypertension in Pediatric CKD

### 2.1. Hypertension in Children and Adolescents

Pediatric hypertension is defined based on normative distributions. It is estimated that 3.3–4.8% of children and adolescents have hypertension and 7.3–12.4% have prehypertension [3,4]. However, an evaluation of prevalence of pediatric hypertension is challenging on a global scale, in view of various definition in different guidelines [14,15,16]. Compared to 2017 American Academy of Pediatrics (AAP) guidelines which used the adult BP cut-points (≥130/80 mmHg) to define hypertension in adolescents ≥13 years of age [14], the 2016 European Society of Hypertension (ESH) guidelines recommended European adult cut-points for adolescents ≥16 years of age (≥140/90 mmHg) [15]. ESH guidelines do not exclude overweight/obesity, which could impact the range of normal BP values and classify as normotensive children who are referred to as hypertensive by the AAP nomogram. Accordingly, the adoption of AAP reference table results in an overall increase in the prevalence of hypertension [17]. Another guideline from the Hypertension Canada Guideline Committee provides a simpler method based on fixed cut points by endorsing the new AAP reference tables [16]. Considering the discordant positions of the main current guidelines for pediatric hypertension, unmet needs to develop global consensus to implement guidance for clinical utility and prevention program are exceedingly required.

### 2.2. Methods of BP Measurement in Children

In children, BP can be determined by office BP measurement, 24 h ambulatory BP monitoring (ABPM), and home BP monitoring. Office BP nomograms are created from large reference populations, albeit with limitations of reference values for ABPM and home BP monitoring are derived from a single study. According to current guidelines on office BP, three office assessments of BP must be made. Office BP was traditionally measured using mercury sphygmomanometers. Lately, mercury sphygmomanometers have been progressively replaced by oscillometric devices for BP measurement. Significant differences may occur between oscillometric devices, and they are prone to overestimate BP in the 3–10 mm Hg interval in pediatric population [18,19]. To make the diagnosis of hypertension, an elevated BP reading from at least two separate visits are required [14].

ABPM is more reproducible and better related to target organ damage compared to office BP. As suggested by AAP and ESH guidelines, ABPM is particularly useful in white coat and secondary hypertension. ABPM should be performed in secondary or tertiary centers with specific skills and ability to diagnose and treat pediatric hypertension. The identification of BP abnormalities not evident in office BP readings in pediatric CKD has greatly improved following the utilization of ABPM [20]. Nevertheless, there are limited pediatric data on home BP monitoring, especially in CKD.

### 2.3. Hypertension in Pediatric CKD

In childhood CKD, hypertension is the most common comorbidity. One of the first publications from the Chronic Kidney Disease in Children (CKiD) Study was the report of office BP measurements taken at the initial study visit [21]. A total of 54% of children with mild-to-moderate CKD had hypertension. The CKiD study further evaluated BP by ABPM and demonstrated that 83% of the participants had ambulatory hypertension (including increased BP load) [22]. Among them, 37% had masked HTN (normal office BP with abnormal ABPM), 15% had confirmed hypertension (elevated office BP with abnormal ABPM), and 4% had white coat hypertension (elevated office BP with normal ABPM) [22].

Another Cardiovascular Comorbidity in Children with CKD (4C) Study recruiting 737 patients illustrated the discrepancies between office and ambulatory BP. In this study, less than 50% of the children with ambulatory hypertension were diagnosed by office BP, and almost 50% of the patients identified as hypertensive by office BP assessments were found normotensive by ABPM [23]. It is noteworthy that even in CKD stage 1, up to 50% of children had hypertension, no matter which methods of BP measurement were used [24,25]. Similar findings have been reported in other studies [26,27], indicating that a substantial percentage of children with CKD has either undiagnosed or undertreated hypertension. The most important devastating effect of hypertension is on the CV system. It may leave significant footprints in children with CKD that can be carried over to adulthood CVD.

**Figure 1:**
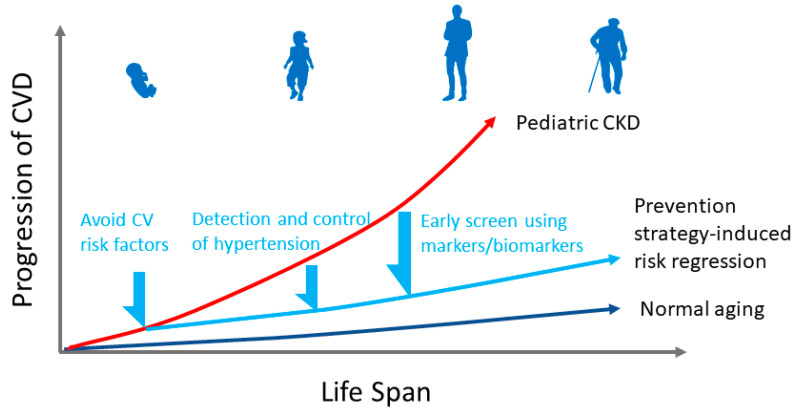
Concept of normal, pediatric chronic kidney disease (CKD) and prevention strategy-induced cardiovascular (CVD) across the lifespan. Avoid cardiovascular risk factors, detection and control hypertension, and screening using markers/biomarkers early in life allows for timely detection of subclinical CVD and initiation of treatment strategies to avert CVD in later life.

In the context of CVD prevention, the first option is to prevent CVD before it occurs [28]. All CV risk factors should be avoided early in life. Given that hypertension is the major CV risk factor, and that hypertension in pediatric CKD is underdiagnosed and undertreated, early detection and control of hypertension is thereby our top priority. Furthermore, screening of CV health by surrogate markers and biomarkers should be performed in childhood in order to detect subclinical CVD and initiate treatment strategies. Importantly, the reversibility of CV risk appears to be optimal in childhood rather than adulthood. The concept of CVD prevention in pediatric CKD is depicted in Figure 1.

## 3. Cardiovascular Risks and Biomarkers in Pediatric CKD

### 3.1. Cardiovascular Risk Factors in Pediatric CKD

In adults, numerous traditional and non-traditional risk factors are widely known and have been identified in CKD population. A meta-analysis study identified 29 routinely collected risk factors of which aging, male gender, hypertension, diabetes mellitus, hyperlipidemia, smoking, left ventricular hypertrophy, albumin, phosphate, urate and hemoglobin were found to be statistically significant in their association with future CV events in adult CKD [29]. In addition, several traditional risk factors related to CVD in children might also be applied to pediatric CKD population [30]. Moreover, several risk factors for congenital anomalies of the kidneys and urinary tracts (CAKUT) [31], the leading causes of pediatric CKD, are also related to CVD and hypertension later in life [31,32]. These risk factors include prematurity, low birth weight (LBW), maternal illness, and male gender. The traditional and non-traditional risk factors in pediatric CKD are included in Table 1.

As overt CVDs are rare at a young age, it is hard to achieve hard end points of CV events and establish the CV risk in pediatric population. Thus, surrogate markers of subclinical CVD and biomarkers of CVD progression have been utilized in this population [33,34]. Some of the most frequently established markers/biomarkers will be described as follows in more detail.

### 3.2. Structural and Functional Markers in Pediatric CKD

So far, various pediatric studies have assessed structural and functional markers to evaluate CV risk such as ABPM, the carotid artery intima-media thickness (cIMT), flow-mediated dilatation (FMD), augmentation index (AI), pulse wave velocity (PWV), ambulatory arterial stiffness index (AASI), left ventricular mass index (LVMI), etc.

#### 3.2.1. ABPM

Hypertension is closely linked to subclinical CVD in children with CKD [35]. Pediatric research in CKD indicates that hypertension is linked to the presence of abnormal surrogate markers of CVD including increased cIMT, PWV and LVMI [35]. Early diagnosis of BP abnormalities by ABPM in pediatric CKD is recommended by the AAP and ESH guidelines [14,15], as nocturnal hypertension, masked hypertension, and abnormal BP loads those are not detectable with office BP assessments.

In children with CKD, masked hypertension increases the risk for left ventricular hypertrophy (LVH) [26]. The CKiD study showed that nocturnal hypertension is significantly associated with risk of kidney replacement therapy [36]. Data from the 4C study also revealed that nocturnal hypertension was associated with increased cIMT, PWV, and LVH [37]. There are many children with CKD who have normal BPs on ABPM, but elevated BP loads (>25%) [26,35]. However, whether elevated BP loads predict either target organ damage or CV events independently of ambulatory BP remains unclear, not only in children but also in adults [38,39].

#### 3.2.2. Carotid Artery Intima-Media Thickness

The utilization of ultrasound diagnostic tests of carotid intima-media thickness (cIMT) as a marker of large artery structure has been applied to pediatric CKD [23,40]. Though the association of cIMT and childhood BP has been confirmed in a systemic review, there remains lack of recommendation for cut-off values [41]. In children, an increase in cIMT has been noted in pre-dialysis CKD stages 2–4 [23,40]. Additionally, the increased cIMT was related to higher BPs and greater LVMI in children with CKD [34,42]. Despite the fact that several reports establishing normative data in children are accessible in the statement, normal references and standardized protocols for assessment of cIMT are still warranted in pediatric population, especially in youths with CKD [43].

#### 3.2.3. Flow-Mediated Dilatation

Endothelial dysfunction is an early predictor of atherosclerosis. The ultrasound method for assessment of endothelial function has been established by determining flow-mediated dilatation (FMD) of the brachial artery [43]. Later studies have demonstrated that FMD was evaluated to assess endothelial dysfunction, a state that entails less local bioavailability of nitric oxide (NO) [44]. The ultrasound measurement of endothelial function in children was first described in 1992 [45]. Since then, FMD has been applied to predict CV risks in children with CKD [46,47,48]. A previous study reported that mean baseline diameter of the brachial artery was significantly greater, and mean flow-mediated vasodilation was decreased [46]. Additionally, reduced brachial artery FMD was accompanied by high BPs and cIMT in children with pre-dialysis CKD [47]. Nevertheless, another study recruiting 125 children with CKD stages 1–4 showed that FMD did not correlated with NO-related biomarkers, BP load, and other CV surrogate markers [48]. Hence, further longitudinal studies are needed before FMD might prove valuable in determining the true benefits of early prediction of CVD in pediatric CKD.

#### 3.2.4. Arterial Stiffness

Arterial stiffness is a sign of functional and structural alterations of arterial wall integrity. As arterial stiffening can precede development of hypertension, the evaluation of large artery stiffness has become a recommended vascular marker as a surrogate end point for CV risk assessment [49]. Arterial stiffness is usually measured by use of tonometric devices as an estimate of aortic PWV using the carotid and femoral sites. PWV is presently the gold standard of arterial stiffness assessment. However, its use in children is challenging because of technical difficulties and the poor standardization between algorithms for calculating PWV [50]. So far, several studies have reported increased PWV in children with different stages of CKD [37,48,50,51,52]. Augmentation index (AI) is another indicator of arterial stiffness derived from the ascending aortic pressure waveform. Similar to PWV, children with CKD had increased AI [52,53]. Furthermore, the ambulatory arterial stiffness index (AASI) is an indirect arterial stiffness index, which has proven to be associated with adverse CV events [54]. The AASI can be simply calculated from 24-h ABPM by computing the regression slope of diastolic BP on systolic BP. The AASI was defined as 1 minus the regression slope. Prior research in pediatric CKD indicated that elevated AASI is linked to high BP [55,56].

#### 3.2.5. Left Ventricular Mass Index

All pediatric guidelines suggest echocardiography at the time of confirmed hypertension [57]. In children, LVH was defined as a LVMI by indexing left ventricular mass allometrically to height to the 2.7 power [58]. In pediatric CKD, LVH develops relatively early and becomes more prevalent as kidney function declines [27,59]. Approximately 20 to 50% of children with CKD stages 2–4 have LVH [27]. Systolic BP is the most critical independent risk factor for LVH in children with CKD [23,60,61]. Conversely, the use of antihypertensive medications can lead to a decreased frequency of LVH with normalization of BP [62]. Progressive LVH causes disturbances in ventricular conduction and ventricular function. Even in pediatrics, this ongoing chamber dilation predisposes these children with CKD to cardiac dysfunction and sudden death [27].

### 3.3. Biomarkers of CVD in Pediatric CKD

**Figure 2:**
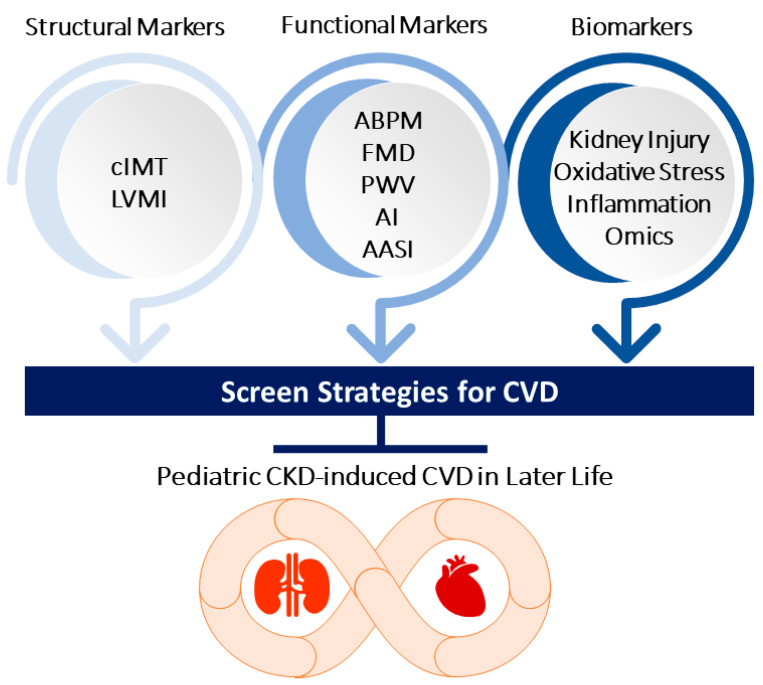
Screen strategies used for prevention of pediatric chronic kidney disease (CKD)-induced cardiovascular (CVD) include structural markers, functional markers, and biomarkers. cIMT = carotid intima-media thickness; LVMI = left ventricular mass index; ABPM = 24 h ambulatory BP monitoring; FMD = flow-mediated dilatation; PWV = pulse wave velocity; AI = augmentation index; AASI = ambulatory arterial stiffness index.

Currently, many biomarkers assessed in the blood and urine have been identified in CKD, and they not only reflect the underlying pathophysiological processes of kidney damage but also CV risk [63,64]. Only a few of them have been tested in pediatric CKD [65,66], especially focusing on hypertension and CV risk. We explored the common mechanisms of CVD in CKD and the pathophysiologic processes of endothelial dysfunction, kidney injury, inflammation, and oxidative stress that are hypothetically classified by specific biomarkers. Furthermore, we summarized reported omics-related biomarkers for CVD in pediatric CKD research. Figure 2 illustrates a brief summary of prevention strategies used for screening CVD in pediatric CKD.

#### 3.3.1. Biomarkers of Endothelial Dysfunction

Endothelial dysfunction begins in the early stages of CKD and progresses with kidney disease severity [67], suggesting that it may be the important mechanism linking CKD to the increased CV risk [68]. The mechanisms behind endothelial dysfunction in CKD include reduced NO production due to reduced L-arginine levels, increased oxidative stress, increased NO synthase inhibitors asymmetric dimethylarginine (ADMA), symmetric dimethylarginine (SDMA), etc. [69,70,71].

A previous report showed that ADMA and SDMA were high in children with CKD, and intermediate NO metabolites were low [72]. It has long been known that L-arginine is the substrate for NO production and our body can use L-citrulline to make L-arginine [73]. In CKD, renal L-citrulline uptake is reduced, the amount of L-citrulline converted to L-arginine in the kidney is diminished, and plasma L-citrulline levels and turnover are increased [74]. In children with CKD stages 1–3, a high plasma ratio of L-citrulline-to-L-arginine significantly correlated with abnormal ABPM profile, including nocturnal hypertension, increased diastolic BP load, and nocturnal BP non-dipping [75]. Another study indicated that the plasma ratio of L-arginine-to-ADMA, an index of NO bioavailability [76], was elevated in children with CKD stages 2–3 and positively correlated with BP. These findings suggest that the increase in this ratio reflects a compensatory response to elevation of BP in children with early stages of CKD [77]. Several studies found plasma ADMA levels were elevated in children with pre-dialysis CKD [78,79,80]. Additionally, elevated plasma ADMA levels occur in parallel with increased BP loads [75] and AI [77].

In addition to testing plasma, some studies evaluating urinary ADMA concentrations in children with pre-dialysis CKD are currently in progress [81,82,83]. In pediatric CKD studies from our group [82,83], we observed that urinary ADMA level alone was not related to CV risks. However, the combined ratio between ADMA and SDMA, offers a better correlation with BP load in children with early-stage CKD.

#### 3.3.2. Biomarkers of Kidney Injury, Oxidative Stress and Inflammation

Understanding the molecular mechanisms that cause renal inflammation is an important approach for identifying early targets for prevention of kidney injury progression to CKD [84]. Although a number of urinary biomarkers, implicated in kidney injury and inflammation, were reviewed elsewhere [84,85], very few of them have been examined in pediatric CKD.

Kidney injury molecule-1 (KIM-1) is a transmembrane glycoprotein expressed in proximal tubular cells and is related to the development of renal fibrosis [86]. The CKiD study showed plasma KIM-1 was higher in children with non-CAKUT CKD as compared to those with CAKUT [87]. Importantly, plasma KIM-1 was independently associated with the risk of CKD progression [87]. Increasing evidence suggests KIM-1 is a promising, new biomarker for early diagnosis and prediction of clinical outcome in CVD [88]. However, further research in the predictive value of KIM-1 is still needed in pediatric CKD for CV risk.

In adults, several inflammatory biomarkers have been identified to predict CV events in CKD [89]. The most frequently used biomarkers were C-reactive protein (CRP), interleukin-1 (IL-1), interleukin-6 (IL-6), tumor necrosis factor-α (TNF-α), and TNF-α receptor type 1 (TNFR1) and type 2 (TNFR2). Interleukin-6 is an inflammation protein biomarker produced by the liver, which has been used as a potential predictor of all-cause and CV mortality in the adult CKD population [90]. It is shown that IL-6 is a better biomarker than acute phase proteins (e.g., CRP and TNF-α) for mortality prediction [91]. In pediatric CKD, plasma TNFR1 and TNFR2 have been associated with CKD progression [87,92]. A recent study revealed that plasma levels of TNF-α and monocyte activation marker soluble CD14 were higher in children with CKD stages 3–4 than controls [93]. However, their ability to improve clinical CV risk prediction in childhood CKD is still unexplored.

In addition, oxidative stress has a key role in the pathogenesis of CVD as well as CKD [94,95], therefore, it is crucial to explore oxidative stress markers to predict CV events in CKD patients [96]. Most biomarkers of oxidative stress related to CV risks in adult CKD have not been evaluated in pediatric CKD [97,98]. So far, only one cross-sectional study recruiting 65 children with CKD stages 1–5 showed serum oxidized low-density lipoprotein levels correlated with LVH and hypertension [99].

Moreover, the elevation of fibroblast growth factor 23 (FGF23) levels is noted in CKD patients [100]. Current research has demonstrated that FGF23 not only severs as a marker of mineral and bone disease (MBD), but is also implicated in inflammation and kidney injury [100,101]. The CKiD study demonstrated that plasma FGF23 is associated with CKD progression [102]. Importantly, several pediatric studies indicate that FGF23 is associated with the development of LVH and thereby could be a CVD risk factor in pediatric CKD [103,104,105].

#### 3.3.3. Omics-Related Biomarkers

Omics technologies have been applied to universal detection of genes, mRNA, proteins and metabolites in kidney disease [106]. Although several genes, including uromodulin (UMOD), shroom family member 3 (SHROOM3) and engulfment and cell motility 1 gene (ELMO1) have been strongly associated with CKD [107], their roles in hypertension and CV outcome are still unclear. So far, the largest pediatric CKD genomic study recruiting 1136 children was conducted by the international Pediatric Investigation for Genetic Factors Linked with Renal Progression (PediGFR) Consortium to identify common genetic variants in pediatric CKD [108]. Several single-nucleotide polymorphisms (SNPs) were identified in six regions for CKD, but none of them were associated at genome-wide significance.

Using transcriptomic approach, nine genes IFI16, COL3A1, ZFP36, NR4A3, DUSP1, FOSB, HBB, FN1, and PTPRC have been identified as significantly differentially expressed both in diseased glomeruli and tubules by an integrative bioinformatics analysis from over 250 Affymetrix microarray datasets [109]. Whilst these omic markers are facing the same problem, they need to be further validated for CKD progression and CVD risk in pediatric population.

In the past decade, urine proteomic mass spectrometry (MS)-based approaches have provided valuable information about protein contents and urinary peptide in CKD with different etiologies [110]. So far, several disease-specific biomarkers have been identified, such as CD44 in membranous nephropathy, dipeptidase 1 (DPEP1) and apolipoproteins in focal glomerulosclerosis (FSGS), and the laminin G-like 3 (LG3) fragments of endorepellin in IgA nephropathy [110,111]. Although many studies have been carried out in adults, proteomic analysis of CKD has not widespread transferred into the pediatric realm [111,112]. Using isobaric tags for relative and absolute protein quantification (iTRAQ)-based proteomic analysis, one study identified 20 differentially expressed proteins associated with hypertension in children with CKD stages 1–4 [113]. Among them, children with CKD stages 2–4 had a higher plasma apolipoprotein II (ApoC-II) level than those with CKD stage 1. Additionally, ApoC-II was correlated with LVH and an abnormal ABPM profile [114]. In a consequent study, another proteomic biomarker complement factor H (CFH) and related proteins were studied in 102 children with CKD stages 1–4 [115]. Plasma CFH-related protein-2 (CFHR2) level was higher in children with CKD and ambulatory hypertension. Moreover, CFH-related protein-3 (CFHR3) was negatively correlated with LVH [115]. These findings suggest that early assessment of above-mentioned proteomic biomarkers may have clinical utility in discriminating CV risk in children with pre-dialysis CKD.

Along with proteomics, metabolomics also affords a great insight into disease mechanisms. Accordingly, metabolomics has been used to search potential biomarkers [116]. In patients with CKD, metabolomic profiling identified several metabolites associated with alterations in carbohydrates, amino acids, nucleotides, and lipids metabolism [116]. As CKD progression is concomitant with altered metabolism, a number of metabolites are being studied as potential biomarkers [64,116]. Untargeted metabolomics for biomarker discovery has been applied in pediatric CKD [117,118,119,120,121]. In early stage of CKD, one metabolic study revealed that sphingosine-1-phosphate, cis-4-decenoylcarnitine, n-butyrylcarnitine, and aminoadipic acid were increased in children with CKD, while bilirubin was decreased [117].

The CKiD study also applied untargeted metabolomics to recognize 825 metabolites in children with CKD [119,120]. Among them, seven metabolites were significantly associated with CKD progression, including lanthionine, N6-carbamoylthreonyladenosine, 5,6-dihydrouridine, C-glycosyltryptophan, pseudouridine, 2-methylcitrate/homocitrate, and gulonate [119,120]. Another metabolomics study enrolling 2086 children with CKD revealed that CKD was significantly associated with phenylalanine/glycine, citrulline, acylcarnitines, acylcarnitine ratios, etc. [121]. Most metabolic studies in pediatric CKD are mainly focused on identifying biomarkers for CKD progression [122]. There was only a few pediatric CKD research using targeted metabolomics approach to explore biomarkers of CVD risk.

In CKD, uremic toxins have a crucial role in CV morbidity and mortality [123]. Trimethylamine-N-oxide (TMAO) is a uremic toxin as well as a potential causal factor for CVD [124]. Gut microbiota metabolize dietary choline and carnitine to generate trimethylamine, the precursor of TMAO. In a cross-sectional study recruiting 115 children with CKD stages 1–4 showed children with CKD stages 2–4 had greater plasma TMAO and TMA concentrations compared with those with CKD stage 1 [25].

**Figure 3:**
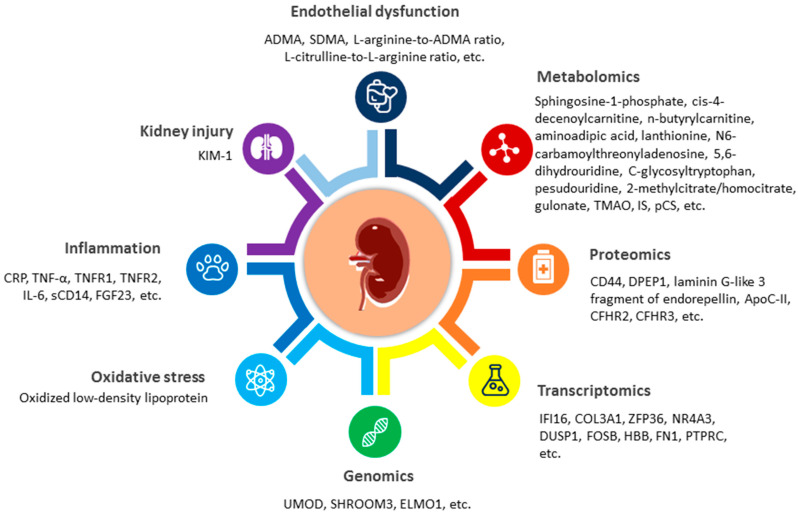
Different types of potential biomarkers in pediatric chronic kidney disease (CKD) for cardiovascular (CVD) risk. ADMA = asymmetric dimethylarginine; SDMA = symmetric dimethylarginine; KIM-1 = kidney injury molecule-1; CRP = C-reactive protein; IL-6 = interleukin-6; TNF-α = tumor necrosis factor-α; TNFR1 = TNF-α receptor type 1; TNFR2 = TNF-α receptor type 2; sCD14 = soluble CD 14; FGF23 = fibroblast growth factor 23; UMOD = uromodulin, SHROOM3 = shroom family member 3; ELMO1 = engulfment and cell motility 1 gene; DPEP1 = dipeptidase 1; ApoC-II = apolipoprotein II; CFHR2 = complement factor H-related protein 2; CFHR3 = complement factor H-related protein 3; TMAO = trimethylamine-N-oxide; IS = indoxyl sulfate; pCS = p-cresil sulfate.

Additionally, several gut microbiota-derived tryptophan metabolites are recognized as uremic toxins, such as indoxyl sulfate (IS), p-cresil sulfate (pCS) and indoleacetic acid (IAA) [123,125]. Considering the complexity of tryptophan metabolic pathways, various tryptophan-derived metabolites are involved in the pathophysiology of CKD and hypertension [125]. In adult CKD, these uremic toxins are implicated with CVD and have clinically been correlated with CVD motility and mortality [126]. In pediatric CKD research, only the 4C study demonstrated that IS and pCS had negative associations with renal function [127]. Notably, IS was tightly linked to a higher cIMT and progression of PWV within 12 months, independent of other risk factors [127]. A summary of the potential biomarkers in pediatric CKD for CVD risk is given in Figure 3.

At the moment, an ideal biomarker for CKD does not exist and the overlap between the biomarkers is a reality [128,129]. A panel of biomarkers that covers the entire pathogenic process of CKD might optimize the specific value of each biomarker [130]. A recent study used machine learning to identify metabolomic signature panels for detecting metabolic sub-pathways associated with different CKD causes in pediatric patients [131]. In recent years, composite biomarkers panels have much improved sensitivity and specificity than individual biomarker for early detection and prediction of disorders. In the near future, using panels of biomarkers could probably improve the identification and prediction of CV risk for children with CKD in the era of precision medicine.

## 4. Special Considerations for Pediatric CKD

Despite promising results obtained in identification of biomarkers for CV risk assessment in adults, the efforts in pediatric CKD are still insufficient. Children are unique in that they are undergoing structural and functional changes in the kidney throughout childhood, a process that can substantially impact their CKD progression and risk of CKD management. As a population with rare CV events, CKD youth potentially receive substantial benefits from aggressive attempts at the early prevention of CVD. Accordingly, pre-dialysis CKD in children is the optimal time to both identify risk factors and intervene in an effort to prevent future CVD.

There are several distinct differences between pediatric and adult CKD populations, such as comorbidity patterns and underlying causes of CKD. Studying and developing markers/biomarkers of pediatric CKD has several challenges. This maturation of cardiovascular and kidney function needs to be considered. Large pediatric CKD studies are therefore warranted to establish age-dependent normal reference range values for structural and functional markers as well as biomarkers for CV risk assessment in different age groups. Next, CKD in children is most commonly due to CAKUT, in contrast with adults where they frequently have various risk factors for CKD such as hypertension and diabetes which may confound results and interpretation of biomarkers. Hence, focused research on CKD in children with different groupings (i.e., CAKUT vs. non-CAKUT) to adults is desired. Hypertension is the most common comorbidity in pediatric CKD [21,22,23], even in CKD stage 1 [24,25]. Despite the recent advances in developing biomarkers in pediatric CKD, most of them are not yet evaluated in the context of hypertension and CV risk. Furthermore, one in ten babies are born preterm [132]; these babies have a higher risk for CKD and CVD due to low nephron endowment [133]. Although the role of low nephron number in developing CKD and hypertension later in life has been extensively studied in animal research [134], potential biomarkers in CV risk assessment in this specific population still await further research.

## 5. Conclusions and Perspectives

Hypertension is highly underdiagnosed and undertreated in pediatric population, even in CKD. Efforts to avoid known maternal adverse exposures and promote optimal healthy behaviors in early childhood could have a significant impact on preventing hypertension in CKD youth. As the most important adverse effect of hypertension is on CVD, developing and validating reliable functional and structural markers, and biomarkers, for CV risk is thereby urgently required in pediatric CKD. Considering the complexity of CVD and CKD, it is unlikely that one biomarker will best predict CVD in pediatric CKD. Hence, there is an additional need to further develop combined biomarker panels with increased specificity and sensitivity for further pediatric CKD research.

## Figures and Tables

**Table 1:** Risk Factors for Cardiovascular Disease in Children with Chronic Kidney Disease.

Traditional Factors	Non-Traditional Factors
Family history of atherosclerosis	Left ventricular hypertrophy
Age	Hypoalbuminemia
Male gender	Hyperuricemia
Behavioral/lifestyle	Hyperphosphatemia
Nutrition/diet	Anemia
Physical inactivity	Inflammation
Smoking	Oxidative stress
Hypertension	Endothelial dysfunction
Hyperlipidemia	Prematurity
Obesity	Low birth weight
Diabetes	Maternal illness

## Data Availability

All data are contained within the article.
